# Erectile Dysfunction as an Obesity-Related Condition in Elderly Men with Coronary Artery Disease

**DOI:** 10.3390/jcm13072087

**Published:** 2024-04-03

**Authors:** Małgorzata Biernikiewicz, Małgorzata Sobieszczańska, Ewa Szuster, Anna Pawlikowska-Gorzelańczyk, Anna Janocha, Krystyna Rożek-Piechura, Agnieszka Rusiecka, Jana Gebala, Paulina Okrzymowska, Dariusz Kałka

**Affiliations:** 1 Men’s Health Centre in Wroclaw, 53-151 Wroclaw, Poland; malgorzata@biernikiewicz.pl (M.B.); janagebala@aol.com (J.G.); 2 Clinical Department of Geriatrics, Wroclaw Medical University, 50-369 Wroclaw, Poland; malsobie100@gmail.com; 3 Obstetrics and Gynecology Department, Wroclaw Medical University, 50-556 Wroclaw, Poland; ewa.szuster8@gmail.com; 4 Cardiosexology Students Club, Wroclaw Medical University, 50-368 Wroclaw, Poland; anna.pawlikowska96@gmail.com; 5 Faculty of Medicine, Wroclaw University of Science and Technology, 50-370 Wroclaw, Poland; janochaanna5@gmail.com; 6 Faculty of Physiotherapy, Wroclaw University of Health and Sport Sciences, 51-612 Wroclaw, Poland; krystyna.rozek-piechura@awf.wroc.pl (K.R.-P.); paulina.okrzymowska@awf.wroc.pl (P.O.); 7 Statistical Analysis Centre, Wroclaw Medical University, 50-367 Wroclaw, Poland; agnieszka.rusiecka@umw.edu.pl

**Keywords:** obesity, overweight, erectile dysfunction, sexual dysfunction, prevalence, testosterone, androgens

## Abstract

**Background**: This cross-sectional study aimed to investigate the prevalence of erectile dysfunction (ED) in elderly men with overweight or obesity and coronary artery disease. **Methods**: Patients recruited in cardiac rehabilitation centers post-myocardial infarction provided demographic and anthropomorphic data. ED was assessed using the abbreviated International Index of Erectile Function 5 (IIEF-5) Questionnaire. **Results**: The study included 661 men with a mean age of 67.3 ± 5.57 years, a mean BMI of 27.9 ± 3.6 m/kg^2^, and a mean waist circumference of 98.9 ± 10.23 cm. Over 90% of men experienced ED, with similar proportions across BMI categories. The development of ED in men with a waist circumference of ≥100 cm had 3.74 times higher odds (OR 3.74; 95% CI: 1.0–13.7; *p* = 0.04) than in men with a waist circumference of <100 cm. Men with obesity and moderate-to-severe and severe ED were older compared to those without these disorders (67.1 ± 5.29 vs. 65.3 ± 4.35; *p* = 0.23). **Conclusions**: The prevalence of ED in men with coronary artery disease surpasses 90%. An increased body weight raises the risk of ED, with waist circumference proving to be a more reliable predictor of this risk compared to BMI. Physicians are encouraged to screen elderly patients with cardiovascular disease for ED and address obesity to enhance overall health.

## 1. Introduction

Obesity has been deemed an epidemic due to the rapid increase in the percentage of people with abnormally increased body weight. The rise in obesity began in the US between 1976 and 1980 and subsequently spread across Westernized countries [1,2]. Obesity is a complex condition with a significant impact on mortality and morbidity. The Global Burden of Disease Study reported that 4.7 million deaths were directly linked to obesity in 2017 [3], leading to increased societal costs and a decreased health-related quality of life. The burden of obesity results in adverse consequences not only on health. It also contributes to psychological problems, depression, stigmatization, disability, and productivity loss at work [4,5,6].

Body mass index (BMI) has been widely used for screening increased body weight. This index is a numerical value calculated from a person’s weight, in kilograms, and height, in meters. BMI values over 25 kg/m^2^ indicate that an individual’s weight is higher than what is considered healthy for his/her height. This condition raises the risk of various health issues associated with excess body weight [2,7]. Obesity and being overweight, similarly to other chronic diseases, are considered lifestyle diseases. Furthermore, obesity is a widely recognized independent predictor of cardiovascular disease, which is strongly related to organic erectile dysfunction (ED) [8,9]. Obesity and ED also share common risk factors such as diabetes, hypertension, physical inactivity, smoking, dyslipidemia, and poor diet [8,9]. Data from the literature confirms that men with abnormally increased body weight complain about health issues associated with their sexual health more often. The meta-analysis conducted by Pizzol et al. [10] investigated the link between BMI, waist circumference, and ED. Based on 45 articles involving 42,489 men, the chance of experiencing ED was significantly higher in overweight men (odds ratio [OR] 1.31; 95% confidence interval [CI]: 1.13–1.51) and even higher in men with obesity (OR 1.60; 95% CI: 1.29–1.98). The occurrence of ED was also linked to a higher BMI (mean difference [MD] 0.77; 95% CI: 0.57–0.97 kg/m^2^) and a larger waist circumference (MD 5.25; 95% CI: 1.3–9.21) [10]. In another meta-analysis, Li et al. [11] studied how losing weight affects erectile function in men who are overweight or obese. The analysis of 5 studies involving 619 participants revealed a significant weight loss in men undergoing the weight loss intervention, compared to the control group (MD −18.07 kg; *p* < 0.01). A significant BMI difference of −9.6 kg/m^2^ (*p* < 0.01) was also reported. Moreover, the improvement in the International Index of Erectile Function (IIEF) was noteworthy at 1.99 (*p* < 0.01), suggesting that weight loss may serve as adjunctive therapy for ED in men who are overweight or obese [11].

**Figure 1:**
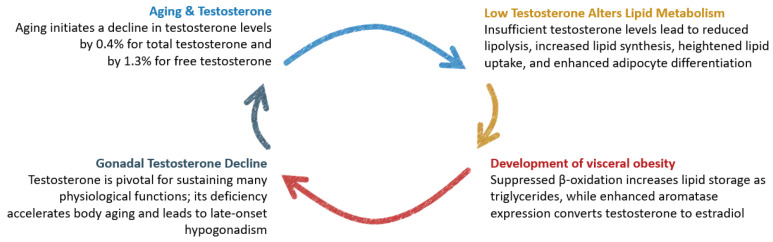
Domino effect of testosterone decline.

Clinical data linking obesity and ED are evident in the biochemical pathways, where testosterone plays a crucial role in mediating this relationship. The definition of hypogonadism in men pertains to testicular failure associated with androgen deficiency in the context of the physiology of the hypothalamus–pituitary–testis axis, which changes across various stages of a man’s life [12,13]. The analysis of data from the European Male Aging Study showed that concentrations of testosterone drop progressively by 0.4% for total testosterone and by 1.3% for free testosterone per year. Furthermore, a decrease in free testosterone level was significantly higher in men with obesity (by 5.09 nmol/L; *p* < 0.001), in comparison to normal-weight men [14]. The decreased testosterone levels during aging impact lipid metabolism in cells. Free fatty acids, either released into the bloodstream or obtained from lipoproteins, can be utilized for energy or stored as triglycerides within cells. When testosterone levels are insufficient, there is a reduction in lipolysis, an increase in lipid synthesis, heightened lipid uptake, and enhanced adipocyte differentiation. Additionally, lipids that would typically be broken down during the β-oxidation process are more likely to be stored as triglycerides than utilized for energy. Consequently, hypogonadal men experience a decrease in lean body mass and an increase in fat mass, particularly in the abdominal or central region [15]. Moreover, adipocytes exhibit a high expression of aromatase, an enzyme responsible for converting testosterone to estradiol. This enzymatic process leads to a decrease in circulating androgens. Simultaneously, estrogens, produced as a result of this process, function as part of a negative feedback mechanism on the hypothalamic–pituitary axis. This action suppresses gonadotropin-releasing hormones and, subsequently, luteinizing hormones, ultimately contributing to a decline in gonadal testosterone release [16]. The escalation of body weight fuels a vicious cycle by directly diminishing testosterone levels, thus reinforcing the development of obesity. The interplay of dependencies between the impact of aging on testosterone levels, the development of obesity, and the consequent negative effects on health is illustrated in Figure 1.

While the connection between obesity and ED is evident clinically and pathophysiologically, there are gaps in the current knowledge regarding real-world data on the strength of the relationship between body weight and the occurrence of ED. To fill this gap, we conducted a cross-sectional study to examine the prevalence of ED in elderly men who are overweight or obese and have comorbid coronary artery disease, considering accompanying modifiable risk factors. The evidence regarding the prevalence of ED in men who are obese or overweight is limited. The novelty of this study lies in providing robust data on the prevalence of ED among elderly men with cardiac conditions and elevated body weight.

## 2. Materials and Methods

Patients were recruited from cardiac rehabilitation centers while undergoing rehabilitation following an event of myocardial infarction. All were diagnosed with coronary artery disease. Demographic data and details regarding modifiable risk factors were gathered through questionnaires. Clinical data were obtained from medical records. The intensity of physical activity was assessed using a form modeled on the Framingham questionnaire, following the instructions of the original questionnaire [17]. A standard of 1000 kcal per week was established as the minimum intensity for leisure-time physical activity, aligning with efforts to prevent cardiovascular diseases as a primary intervention [18]. To assess the occurrence of ED, we utilized an abbreviated International Index of Erectile Function 5 (IIEF-5) Questionnaire [19]. This questionnaire consists of five questions, each scored from 0 to 5. ED was diagnosed when the overall score was 21 or lower.

We included men diagnosed with coronary artery disease who were 60 years old or above, despite the common use of a 65 year threshold and above to define the elderly population. The age of 65 is based on the traditional retirement age in many countries; however, the United Nations consider adults between the ages of 20 and 60 years to be fully productive [20]. Furthermore, individuals with chronic diseases, particularly cardiovascular disease, may be considered elderly at an earlier age of 60 years.

Additional inclusion criteria required participants to have a recorded BMI value and to complete the IIEF-5 questionnaire. Exclusion criteria included previous surgery for prostatic hyperplasia or prostate cancer, repair of the abdominal aorta or iliac arteries, treatment for any vascular event in the central nervous system, injuries to the spine or pelvis, psychiatric disorders, hormonal disorders other than testosterone deficiency, and undergoing dialysis. None of the patients were undergoing a weight reduction intervention.

All patients provided written informed consent to participate in the study. The study received approval from the local Bioethics Committee at Wroclaw Medical University. It was conducted within the framework of the PREVANDRO project, serving as an introduction to targeted cardiosexology education. Initiated in 2011, the project is ongoing, consistently reporting outcomes on ED in cardiac patients.

Data were statistically analyzed using Statistica software version 13.3 (StatSoft, Tulsa, OK, USA) and were presented as means ± standard deviation (SD) for continuous variables, and numbers (%) for categorical variables. The distribution of values was assessed using the Shapiro–Wilk test. Depending on the group characteristics and the number of groups selected for a single comparison, we employed the Mann–Whitney U test, Chi-square test, or the Kruskal–Wallis test with the post hoc median test. Relationships were examined using Spearman’s rank correlation coefficient. The OR and 95% CI were calculated to compare the risks of ED. Statistical significance was interpreted at *p* < 0.05.

## 3. Results

### 3.1. Characteristics of the Study Group

The study included 661 men with a mean age of 67.3 ± 5.57 years, a mean BMI of 27.9 ± 3.6 m/kg^2^, and a mean waist circumference of 98.9 ± 10.23 cm. None of the patients had a BMI below 18.5, so, to facilitate comparison, they were stratified into three BMI groups, as follows: men with normal weight or with weight within a normal range (*n* = 143; 21.6%), men who were overweight (*n* = 344; 52.0%), and men with obesity (*n* = 174; 26.3%). Regarding the occurrence of risk factors and comorbidities, the groups stratified by BMI category did not significantly differ in terms of age, education, severity of ED, dyslipidemia, smoking, and leading a sedentary lifestyle. However, hypertension and type 2 diabetes were more frequent in men with an increased BMI. All patients were on pharmacotherapy, with 95.2% taking statins, 93.2% taking beta-blockers, 81.6% acetylsalicylic acid, 72.2% taking angiotensin-converting-enzyme inhibitors, 42.9% clopidogrel, 41.8% diuretics, 20.7% calcium channel blockers, 10.4% angiotensin receptor blockers, and 6.8% beta-adrenergic blockers. Table 1 presents the characteristics of the study group.

### 3.2. Association between BMI and ED

The correlation between BMI and IIEF-5 score was weak and not significant (r = −0.02; *p* = 0.60). Additionally, no numerical increase in the IIEF-5 score was observed when patients were stratified by BMI categories and five degrees of ED severity. However, there was a slight numerical increase in the percentage of patients classified as having moderate to severe ED; nevertheless, this increase was not statistically significant with *p* = 0.987 (Table 2).

### 3.3. Association between Waist Circumference and ED

**Figure 2:**
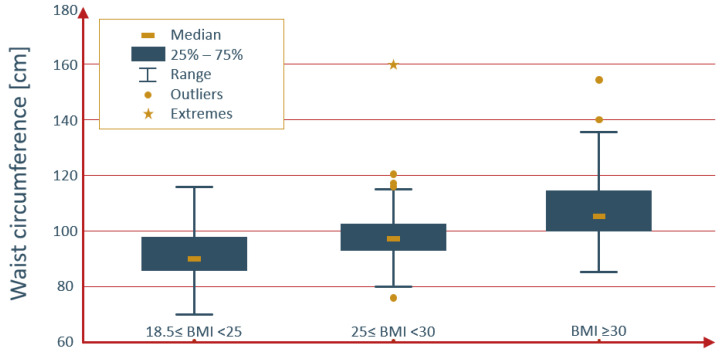
Distribution of waist circumference values by BMI categories.

The correlation between waist circumference and IIEF-5 score was weak but significant (r = −0.20; *p* < 0.0001). The distribution of waist circumference values across BMI categories is illustrated in Figure 2.

A significant numerical rise in the percentage of patients classified as having moderate to severe ED was observed across quartiles of waist circumference (*p* = 0.02) (Table 3).

Additionally, when considering a threshold of 100 cm (75% percentile) for waist circumference, in relation to the occurrence of ED, the OR was 3.74 (95% CI: 1.0–13.7; *p* = 0.04). This means that the odds for the development of ED were 3.74 times higher for men with a waist circumference of 100 cm and above, compared to men with a waist circumference below 100 cm.

### 3.4. Characteristics of Obese Men with ED

To characterize men with obesity and erectile dysfunction, we compared men with a normal weight, who did not report having ED, to men with obesity and who are overweight, with an IIEF-5 score of 21 and below. Although numerical differences were observed in some variables, only the difference in waist circumference reached statistical significance. A detailed comparison of both groups is presented in Table 4.

## 4. Discussion

Our study found that the prevalence of ED is over 90% in elderly men with coronary artery disease, with similar proportions across BMI categories. The correlation between BMI and IIEF-5 score was weak and not significant (r = −0.02; *p* = 0.60), while the correlation between waist circumference and IIEF-5 score was significant (r = −0.20; *p* < 0.0001). Both an elevated BMI and a waist circumference of <100 cm (75% percentile) increased the risk of developing ED. No important differences were found between typical-weight men without ED and men with ED who were obese or overweight, including age; however, the group of men with increased body weight and ED was numerically older by almost 2 years.

In our study, we employed two methods to assess increased body weight, namely BMI and waist circumference. Both are recommended by the European Association of Urology Guidelines on Sexual and Reproductive Health to be taken during the physical examination of all individuals suspected of late-onset hypogonadism [21]. In the literature, there are few publications on how common ED is in men with a high BMI. We found only one study that looked at ED in elderly men based on their BMI. Cho et al. [22] conducted a study with 208 elderly Koreans in a suburban area, all aged 65 or older (average age 67.4 ± 8.2 years). ED was diagnosed using the IIEF-5 questionnaire; however, the cutoff value for ED was a score of 18, in contrast to Rosen et al.’s [19] cutoff value of ≤21 for diagnosing ED. As a result, some men with mild ED were categorized as not having ED, potentially leading to underestimated percentages of men experiencing ED. After excluding sexually inactive men and using the second quintile of BMI (23.2–24.4) as a reference, the authors observed an increased risk of ED in both the first BMI quintile (23.1 and below), with an OR of 14.09 (95% CI: 2.35–84.60) and the fifth BMI quintile (27.1 and above), with an OR of 4.92 (95% CI: 0.96–25.34) [22]. We added a similar threshold of a score of 18 in the IIEF-5 questionnaire in our analysis. It showed a numerical increase in the percentage of men affected by ED across BMI categories, but this increase was not significant.

Other measures of obesity, particularly focused on visceral fat, involve measuring waist circumference and indices derived from waist circumference measurements. The WHO regards waist circumference as a measure of metabolic risk. A waist circumference above 94 cm indicates an increased risk for metabolic complications, while a waist circumference above 102 cm signifies a substantially increased risk of metabolic complications, when taking into account the male population [23]. The experts from the European Group for the Study of Insulin Resistance (EGIR) [24] included central obesity, defined as a waist circumference of 94 cm and over, as part of metabolic syndrome. According to the National Cholesterol Education Program (NCEP), the Expert Panel on Detection, Evaluation, and Treatment of High Blood Cholesterol in Adults [25] provided one threshold for the determination of abdominal obesity as part of metabolic syndrome (>102 cm); however, with the caveat that metabolic syndrome can also develop in patients burdened with multiple risk factors and marginally increased waist circumference (94–102 cm). Our study did not specifically address metabolic syndrome, but the threshold of 100 cm (75% percentile) indicated a significant increase in the risk of ED. In a study on the general population, Cao et al. [26] explored the relationship between the weight-adjusted waist index (WWI) and ED. The WWI was calculated by dividing waist circumference, in centimeters, by the square root of weight, in kilograms. Data for this analysis were obtained from the National Health and Nutrition Examination Survey (NHANES) 2001–2004, comprising 3884 participants, of whom 1056 (27.19%) had a history of ED. This cohort was regarded as a representative sample of noninstitutionalized civilian residents in the US. The study population, with a mean age of 60.62 ± 0.48 years (range: 20 to 85 years) was younger compared to our sample and reflected the characteristics of the general population. While the prevalence of ED was low, their study demonstrated an increase in ED prevalence with rising body weight, 22.98% for a BMI below 25, 26.77% for a BMI of 25 and above but below 30, and 31.95% for a BMI of 30 and higher. The WWI was significantly higher in men with ED compared to those without ED (11.22 ± 0.03 cm/√kg vs. 10.59 ± 0.02 cm/√kg, *p* < 0.001). Additionally, the WWI exhibited a stronger predictive value for ED than BMI and waist circumference. It is worth noting that NHANES used a self-reported single-item Massachusetts Male Aging Study (MMAS) question to classify men with ED, while we employed the IIEF-5 questionnaire, indicating another difference between our studies [26]. Kessler et al. pointed out that the prevalence of ED measured using MMAS tends to produce slightly different levels in comparison to those measured using IIEF questionnaires [27]. To relate to this study, we investigated the correlation between waist circumference and the IIEF-5 score. Waist circumference appeared to be more tightly connected with the IIEF-5 score than BMI, which may indicate that it could serve as a more reliable predictor of ED risk than BMI. Nevertheless, a direct comparison between these studies is not possible due to using different questionnaires for ED.

There is a growing body of evidence that suggests that BMI is not an ideal indicator of excess body weight in the elderly, as confirmed by our analysis. The International Atherosclerosis Society (IAS) and International Chair on Cardiometabolic Risk (ICCR) Working Group on Visceral Obesity underscored that abdominal obesity serves as a risk factor for premature atherosclerosis and cardiovascular disease. The consensus among experts in the group meeting was that relying solely on BMI is insufficient for accurately assessing and managing the cardiometabolic risk linked to higher levels of body fat. This is because BMI does not distinguish between fat distribution patterns and may underestimate the risk associated with central adiposity. Thus, experts advocate for incorporating waist circumference as a routine measurement in clinical practice alongside BMI to more effectively classify obesity [28]. Another significant factor is age-related changes, which encompass the loss of muscle mass, the redistribution of fat, and frailty, all of which are common in older adults [29]. In older people, BMI can either underestimate or overestimate body fat mass. Given that fat deposition in the elderly tends to accumulate intra-abdominally, measuring waist circumference provided a more accurate assessment of obesity, as waist circumference provides a more direct measure of adiposity and can better capture changes in body composition that occur with aging. Sarcopenic obesity is characterized by poor quality and an insufficient amount of muscle mass, leading to functional limitations and disability [30]. BMI alone cannot accurately determine sarcopenic obesity because it does not differentiate between fat mass and lean muscle mass. In elderly people, measuring waist circumference provides a better assessment; however, other methods such as hydrostatic densitometry, bioelectrical impedance, or dual-energy x-ray absorptiometry would more accurately assess the degree of obesity. Despite the limitations of BMI, it remains widely utilized in clinical studies involving elderly populations.

Diabetes is widely recognized as a risk factor for both ED and cardiovascular diseases. In our study, we investigated diabetes as one of the risk factors. Al-Hunayan et al. [31] conducted a study involving 323 men newly diagnosed with type 2 diabetes from primary care centers in Kuwait. The mean age was 41.7 ± 0.6 years, ranging from 21 to 65 years. To diagnose ED, the IIEF-5 questionnaire was used. The study reported that the percentage of men experiencing ED increased with BMI, 21.5% in normal-weight men, 40% in overweight men, and 59.3% in men with obesity [31]. In our study, 23.8% of the study population were diagnosed with diabetes. Furthermore, the percentage of men with diabetes was 14.7% among those with a normal weight and 35.1% among those with obesity. The same trend pertains to the cardiovascular system’s link with obesity. Visceral obesity adversely affects endothelial function and testosterone levels [32]. In our study, the percentage of men with hypertension was 46.2% among those with a normal weight and 70.1% among those with obesity. Finally, an interesting finding considered the fact that obese and overweight men experienced ED at an equivalent age compared to normal-weight men without ED, 65.3 ± 4.35 years vs. 67.1 ± 5.29 years; *p* = 0.23. This finding indicates that an excess of body weight accelerated the development of ED. We did not identify any other study that investigated whether obesity could accelerate the onset of ED. Nevertheless, these findings highlight an escalating burden of risk factors with increasing body weight in our senior population with coronary artery disease.

Several limitations should be kept in mind while interpreting our results. The study cohort comprised older men with coronary artery disease undergoing cardiac rehabilitation. Patients with cardiovascular diseases have an increased risk of developing ED due to shared risk factors for both conditions. For example, in a similar cohort without age limitation having coronary heart disease with a mean age of 60.73 ± 9.20 age, ED was reported in 79.23% of the men [33]. The global prevalence of ED, as reported by Kessler et al. [27], spans a wide range, starting from 13.1% and reaching 71.2%. The summary of IIEF data obtained from men aged 60 to 69 indicates that a range of 18.1% to 94.1% experience some degree of ED. Another limitation is the use of BMI to define obesity. Although it fails to differentiate between body fat and lean mass and does not provide information about the distribution of body mass, it remains very popular among patients and physicians. For this reason, we recommend increasing awareness about the use of waist circumference for determining central obesity, so this measure can be used alongside BMI. Waist circumference has been proven to be useful and is associated with ED [10,34]. Furthermore, this is a cross-sectional study, which means it cannot establish causality, while ED is self-reported by survey participants and is susceptible to bias. Finally, this study pertains to a Polish population of patients with chronic cardiovascular disease. The association between BMI and IIEF-5 scores, as identified in this study, requires further research in different populations before generalization can be considered.

## 5. Conclusions

The prevalence of ED in men with coronary artery disease surpasses 90%. Men with obesity and who are overweight face an increased risk of developing ED; however, it appears that waist circumference is a more reliable predictor of this risk than BMI. Notably, obese and overweight men experienced ED at a similar age. As a proactive measure, physicians are advised to screen all elderly patients with cardiovascular disease for the occurrence of ED and address obesity to enhance their overall health.

## Figures and Tables

**Table 1:** Characteristics of the study group.

Variable	Total	18.5 ≤ BMI < 25	25≤ BMI < 30	BMI ≥ 30	*p* Value
No of patients, *n* (%)	661	143 (21.6%)	344 (52.0%)	174 (26.3%)	
Age, years					
Mean ± SD	67.3 ± 5.57	68.7 ± 5.52	67.1 ± 5.20	67.6 ± 5.26	0.32
Median	66.0	67.0	66.0	66.00	
IQR	63.0–71.0	64.0–73.0	63.0–70.0	62.0–70.0	
Range	60.0–90.0	60.0–90.0	60.0–85.0	60.0–84.0	
Education, *n* (%)					
Higher	127 (19.2%)	118 (19.6%)	68 (19.8%)	31 (17.8%	0.05
Secondary	211 (31.9%)	46 (32.2%)	120 (34.9%)	45 (25.9%)	
Vocational	191 (28.9%)	32 (22.4%)	96 (27.9%)	63 (36.2%)	
Primary	46 (7.0%)	12 (8.4%)	17 (4.9%)	17 (9.8%)	
Missing data	86 (13.0)	25 (17.5%)	43 (12.5%)	18(10.3%)	
Erectile dysfunction *, *n* (%)					
Severe	142 (21.5%)	29 (20.3%)	75 (21.8%)	38 (21.8%)	0.96
Moderate-to-severe	95 (14.4%)	21 (14.7%)	44 (12.8%)	30 (17.2%)	
Moderate	208 (31.5%)	46 (32.2%)	112 (32.6%)	50 (28.7%)	
Mild	154 (23.3%)	34 (23.8%)	81 (23.5%)	39 (22.4%)	
No dysfunction	62 (9.4%)	13 (9.1%)	32 (9.3%)	17 (9.8%)	
Hypertension, *n* (%)					
Yes	382 (57.8%)	66 (46.2%)	194 (56.4%)	122 (70.1%)	0.004
No	89 (13.5%)	29 (20.3%)	39 (11.3%)	21 (12.1%)	
Missing data	190 (28.7%)	48 (33.6%)	111 (32.3%)	31 (17.8%)	
Type 2 diabetes, *n* (%)					
Yes	157 (23.8%)	21 (14.7%)	75 (21.8%)	61 (35.1%)	0.003
No	281 (42.5%)	68 (47.6%)	141 (41.0%)	72(41.4%)	
Missing data	223 (33.7%)	54 (37.8%)	128 (37.2%)	41 (23.6%)	
Dyslipidemia, *n* (%)					
Yes	273 (41.3%)	58 (40.6%)	125 (36.3%)	90 (51.7%)	0.15
No	165 (25.0%)	31 (21.7%)	91 (26.5%)	43 (24.7%)	
Missing data	223 33.7%)	54 (37.8%)	128 (37.2%)	41 (23.6%)	
Smoking, *n* (%)					
Yes	336 (50.8%)	69 (48.3%)	165 (48.0%)	102 (58.6%)	0.97
No	103 (15.6%)	20 (14.0%)	51 (14.8%)	32 (18.4%)	
Missing data	222 (33.6%)	54 (37.8%)	128 (37.2%)	40 (23.0%)	
Sedentary lifestyle **					
Yes	539 (81.5%)	115 (80.4%)	280 (81.45%)	144 (82.8%)	0.81
No	85 (12.9%)	19 (13.3%)	41 (11.9%)	25 (14.4%0	
Missing data	37 (5.6%)	9 (6.3%)	23 (6.7%)	5 (2.9%)	
Waist circumference					
Mean ± SD	98.9 ± 10.23	92.1 ± 8.60	97.6 ± 7.83	107.0 ± 10.42	<0.001
Median	98.0	90.0	97.0	105.0	
IQR	92.0–104.0	86.0–98.0	93.0–102.0	100.0–104.0	
Range	70.0–160.0	70.0–116.0	76.0–160.0	85.0–154.0	

BMI, body mass index; IQR, interquartile range; SD, standard deviation; * Erectile dysfunction was classified using an abbreviated International Index of Erectile Function 5 (IIEF-5) Questionnaire, as follows: severe (5–7 scores), moderate-to-severe (8–11 scores), moderate (12–16 scores), mild (17–21 scores), and no dysfunction (>21 scores); ** A threshold of <1000 kcal/week was established to identify men who are leading a sedentary lifestyle.

**Table 2:** Study group stratified by BMI categories and erectile dysfunction severity.

	18.5 ≤ BMI < 25	25 ≤ BMI < 30	BMI ≥ 30
Moderate-to-severe ED	96 (67.1%)	231 (67.2%)	118 (67.8%)
No ED and mild ED	47 (32.9%)	113 (32.8%)	56 (32.2%)

BMI, body mass index; ED, erectile dysfunction.

**Table 3:** Study group stratified by waist circumference quartiles and erectile dysfunction severity.

	WC Q1	WC Q2	WC Q3	WC Q4
Moderate-to-severe ED	89 (69.5%)	96 (64.4%)	117 (66.1%)	141 (75.8%)
No ED and mild ED	58 (39.5%)	53 (35.6%)	60 (33.9%)	45 (24.2%)

Q, quartile; WC, waist circumference.

**Table 4:** Comparison between normal-weight men without ED and obese and overweight men with ED.

Variable	Normal Weight, No ED (BMI < 25 and IIEF-5 * > 21)	Obesity, Overweight, ED (BMI ≥ 25 and IIEF-5 * ≤ 21)	*p* Value
Number of patients, n	13 (2.7%)	469 (97.3%)	
Age, years			
Mean ± SD	65.3 ± 4.35	67.1 ± 5.29	0.23
Median (IQR)	65.0 (62.0–67.0)	66.0 (63.0–70.0)	
Range	60.0–75.0	60.0–84.0	
Education, *n* (%)			
Higher	3 (25.0%)	81 (19.8%)	0.77
Secondary	5 (41.7%)	154 (37.7%)	
Vocational	4 (33.3%)	144 (35.2%)	
Primary	0 (0.0%)	30 (7.3%)	
Hypertension, *n* (%)			
Yes	9 (90.0%)	285 (96.9%)	0.62
No	1 (22.0%)	53 (15.7%)	
Type 2 diabetes, *n* (%)			
Yes	4 (44.4%)	126 (40.3%)	0.80
No	5 (55.6%)	187 (97.4%)	
Dyslipidemia, *n* (%)			
Yes	6 (66.7%)	192 (61.3%)	0.75
No	3 (33.3%)	121 (38.7%)	
Smoking, *n* (%)			
Yes	6 (66.7%)	243 (77.4%)	0.45
No	3 (33.3%)	71 (22.6%)	
Sedentary lifestyle **			
Yes	9 (75.00%)	381 (86.2%)	0.27
No	3 (25.00%)	61 (13.8%)	
Waist circumference			
Mean ± SD	91.6 ± 8.88	101.1 ± 9.91	<0.001
Median (IQR)	89.0 (86.0–95.0)	100.0 (95.0–105.0)	
Range	83.0–112.0	76.0–160.0	

BMI, body mass index; ED, erectile dysfunction; IQR, interquartile range; SD, standard deviation. * Erectile dysfunction was classified using an abbreviated International Index of Erectile Function 5 (IIEF-5) Questionnaire, as follows: severe (5–7 scores), moderate-to-severe (8–11 scores), moderate (12–16 scores), mild (17–21 scores), and no dysfunction (>21 scores); ** A threshold of <1000 kcal/week was established to identify men who are leading a sedentary lifestyle.

## Data Availability

The data that support the findings of this study are available on request from the corresponding author.
